# Static Magnetic Field-Mediated Parathyroid Xenotransplantation Modulates Lymphocyte Migration: A Potential Immunosuppression-Free Long-Term Treatment for Hypoparathyroidism

**DOI:** 10.3390/cells15070600

**Published:** 2026-03-28

**Authors:** Ahmed Alperen Tuncer, Gülnihal Bozdağ, Ezgi Hacıhasanoğlu, Özge Karabıyık Acar, Fikrettin Şahin, Gamze Torun Köse, Erhan Ayşan

**Affiliations:** 1 Department of Genetics and Bioengineering, Faculty of Engineering and Natural Sciences, Yeditepe University, Istanbul 34755, Türkiye; gulnihal.bozdag@yeditepe.edu.tr (G.B.); fsahin@yeditepe.edu.tr (F.Ş.); gamzekose@yeditepe.edu.tr (G.T.K.); 2 Department of Pathology, Istanbul Training and Research Hospital, Istanbul 34098, Türkiye; ezgi.hacihasanoglu@saglik.gov.tr; 3 Department of Genetics and Bioengineering, Istanbul Okan University, Istanbul 34959, Türkiye; ozge.acar@okan.edu.tr; 4 Department of General Surgery, Yeditepe University Hospital, Istanbul 34718, Türkiye; erhan.aysan@yeditepe.edu.tr

**Keywords:** parathyroid, static magnetic field, immune response, parathormone, lymphocyte, xenotransplantation

## Abstract

**Highlights:**

**What are the main findings?**

- Continuous exposure to moderate-intensity static magnetic fields (SMFs) induces spatial immune sequestration, physically redirecting lymphocytes and macrophages to the graft periphery and preventing destructive infiltration of the parathyroid parenchyma.
- SMF-assisted transplantation significantly suppresses systemic IFN-gamma levels, indicating a targeted downregulation of the Th1-mediated rejection pathway without inducing systemic inflammatory toxicity or cytokine storms.

**What are the implications of the main findings?**

- This biophysical approach provides a robust, non-pharmacological adjuvant strategy that could eliminate or significantly reduce the requirement for lifelong systemic immunosuppression in endocrine tissue transplantation.
- The study establishes a new paradigm for immunoengineering, demonstrating that physical forces can be utilized as precise, non-invasive tools to modulate the immune microenvironment and enhance long-term xenograft viability.

**Abstract:**

Static magnetic fields (SMFs) are underexplored as biophysical tools for transplant immunomodulation. This study investigated a 300 mT SMF as a non-pharmacological adjuvant to enhance graft survival in parathyroid xenotransplantation. Human parathyroid tissues were transplanted into Sprague-Dawley rats (n = 20) across four groups: control (G1), SMF-only (G2), transplantation-only (G3), and SMF-assisted transplantation (G4). Following 30-day continuous SMF exposure, functional and immunological assessments were performed. G4 achieved the highest systemic PTH recovery (*p* = 0.009) without altering intrinsic secretory capacity. Systemic cytokine profiling revealed significant IFN-gamma suppression in G4 (*p* = 0.0024), suggesting downregulation of Th1-mediated rejection pathways. While G2 showed pro-inflammatory increases (TNF-alpha, GM-CSF), G4 maintained baseline levels, confirming biocompatibility. IHC confirmed that SMF exposure sequestered lymphocytes to the graft periphery, preventing the diffuse infiltration observed in G3. In conclusion, continuous SMF exposure modulates the immune microenvironment by altering lymphocyte migration and IFN-gamma signaling. This biophysical strategy provides localized immunoprotection, potentially offering a drug-free alternative to systemic immunosuppression in endocrine tissue transplantation.

## 1. Introduction

Hypoparathyroidism is a clinically significant endocrine disorder defined by insufficient secretion of parathyroid hormone (PTH), leading to impaired calcium and phosphate regulation and subsequent disruption of mineral homeostasis [1,2,3]. The condition most commonly occurs as a postoperative (post-op) complication following thyroidectomy or parathyroidectomy, where inadvertent damage or removal of parathyroid glands results in inadequate PTH production [4,5,6]. Although hypoparathyroidism can also stem from autoimmune or congenital etiologies, postsurgical cases remain the predominant form encountered in clinical practice [7].

PTH plays a central role in maintaining calcium and phosphate balance by regulating renal reabsorption, skeletal mobilization, and intestinal absorption [8]. Its deficiency results in hypocalcemia, which can manifest as muscle cramps, tetany, and seizures and may lead to long-term complications such as soft tissue calcifications and neurocognitive deficits [9]. Current management approaches primarily involve oral calcium and vitamin D supplementation or recombinant PTH analogs. However, these treatments neither restore endogenous hormone production nor mimic the physiological feedback loop of calcium regulation and are frequently associated with adverse effects, poor patient compliance, and significant long-term costs [10].

Parathyroid transplantation offers a promising curative strategy by reestablishing endogenous PTH secretion. Nevertheless, immune rejection of grafted tissue and the necessity for lifelong systemic immunosuppression remain major barriers to its clinical adoption, particularly in the context of xenotransplantation, where interspecies antigenicity is heightened [11,12,13]. The rejection process involves both innate and adaptive immunity: antigen-presenting cells activate T lymphocytes through major histocompatibility complex (MHC) interactions, initiating a cascade of cytotoxic responses against donor tissue [14,15]. CD4^+^ helper T cells contribute to this process by releasing pro-inflammatory cytokines such as IL-2, IFN-γ, and TNF-α, while CD8^+^ cytotoxic T cells mediate direct graft cell apoptosis [16,17,18]. Additionally, natural killer (NK) cells and macrophages amplify early immune responses by recognizing non-self or stressed donor cells [19,20].

To overcome these challenges, various encapsulation techniques have been developed. These involve enclosing the transplanted tissue in biocompatible hydrogels—such as alginate, collagen, or chitosan—that act as semi-permeable immunoprotective barriers, allowing nutrient and hormone exchange while preventing immune cell infiltration [21,22,23]. Despite the potential of these systems to reduce or eliminate the need for immunosuppressants, they have drawbacks. Fibrotic overgrowth, limited oxygen/nutrient diffusion, and eventual graft failure due to immune recognition of encapsulation materials continue to limit their effectiveness [24,25].

Consequently, alternative non-pharmacological approaches to regulate immune cell behavior are currently under investigation. While electric fields have shown the capacity to influence lymphocyte migration in vitro, their invasive application and need for external hardware limit clinical utility [26,27,28,29]. In contrast, static magnetic fields (SMFs) offer a non-invasive, biophysically precise, and tunable method to influence cellular behavior. SMFs can modulate ion transport, intracellular signaling pathways, membrane receptor dynamics, and reactive oxygen species production via mechanisms including Lorentz force interactions, ultimately affecting immune cell activation and migration [30,31,32,33,34,35,36]. SMFs are classified based on field strength, ranging from hypomagnetic (<5 μT) to high-intensity (>1 Tesla) (T), with moderate fields (1 mT–1 T) considered most applicable for biomedical use due to their safety profile and biological efficacy [37,38].

Although multiple in vitro studies have demonstrated SMF-induced effects on immune cells, osteoblasts, and stem cells, there is a lack of in vivo research exploring the role of SMFs in modulating immune responses following parathyroid tissue transplantation [39,40,41,42,43,44,45,46]. The absence of systemic toxicity and the capacity to manipulate cell behavior through purely physical means position SMFs as an attractive tool for next-generation biocompatible transplant engineering.

The novelty of this approach lies in the concept of using a controlled SMF to spatially redirect or modulate lymphocyte migration without the need for immunosuppressive drugs or invasive barriers. Unlike pharmacological agents that broadly suppress immune function and carry significant risk profiles, SMFs could allow localized immunomodulation, effectively creating an “immune-privileged” microenvironment at the graft site. This represents a significant departure from conventional methods that either attempt to encapsulate the graft or rely on systemic immune suppression. By influencing immune cell positioning and activity through endogenous, physical forces, this approach could open a new paradigm in cell and tissue transplantation, particularly in endocrine organs like the parathyroid gland, where long-term viability and hormone secretion are critical.

Furthermore, SMFs are integrated into clinical protocols, are cost-effective, and do not require direct tissue contact, reducing both regulatory complexity and translational barriers. These properties make SMFs a highly appealing candidate for translational immunoengineering, with broad implications not only for hypoparathyroidism but also for solid organ and cellular transplants in general.

In this study, we evaluated the in vivo effects of moderate-intensity SMF exposure on lymphocyte migration, immune modulation, and functional viability of human parathyroid xenografts in a rat model. To our knowledge, this work represents the first in vivo demonstration of utilizing a controlled static magnetic field as a localized biophysical barrier to achieve spatial immune sequestration. By physically redirecting immune cells without the need for systemic drugs or invasive encapsulation, this approach introduces a novel paradigm for enhancing long-term graft survival in endocrine tissue transplantation.

## 2. Materials and Methods

### 2.1. Preparation of Parathyroid Tissues

Human parathyroid tissues were obtained from four patients who underwent parathyroidectomy for primary hyperparathyroidism. Prior to surgery, informed consent was obtained from all patients, and the study was performed in line with the principles of the Declaration of Helsinki. Approval was granted by the Bezmialem Vakıf University Clinical Research Ethics Committee of the Republic of Türkiye for human trials (2013/99) (Clinical ID: NCT02134483). The surgeries took place at Yeditepe University Hospital, Department of Endocrine Surgery. Excised tissues were initially examined histopathologically in the pathology laboratory, confirming the diagnosis of parathyroid hyperplasia. Following excision, the parathyroid glands were immediately transferred to the Yeditepe University Parathyroid and Thyroid Research Laboratory in harvest medium (Dulbecco’s Modified Eagle Medium [DMEM] supplemented with 1000 U/mL penicillin/streptomycin). In the laboratory, the harvest medium was removed, and the tissues were cryopreserved at −80 °C until the day of the animal experiments. On the day of transplantation, cryovials were rapidly thawed in a 37 °C water bath. The recovered tissue was then placed in a sterile Petri dish containing pre-warmed harvest medium and sectioned into uniform fragments of approximately 3 mm^3^ using a sterile #11 scalpel blade. These fragments were then incubated in a 24-well plate (Corning Inc., Corning, NY, USA) with 2 mL of harvest medium per well at 37 °C in a 5% CO_2_ environment for 1 h to ensure stabilization prior to surgical implantation.

### 2.2. Experimental Setup

For all experimental groups, the following parameters were evaluated to assess the effect of the static magnetic field on graft functionality and immune modulation over 30 days. We evaluated preoperative (pre-op) and postoperative (post-op) serum parathyroid hormone (PTH) levels, serum cytokine levels, quantitative ex vivo PTH secretion from grafted tissues, and immunohistochemical (IHC) analysis of grafts for PTH and lymphocyte markers (CD3, CD20, and CD68). Approximately 3 mm^3^ of parathyroid tissue was transplanted per animal.

### 2.3. In Vivo Parathyroid Tissue Xenotransplantation and Magnet Placement

All animal experiments were conducted at Yeditepe University in accordance with the National Research Council’s Guide for the Care and Use of Laboratory Animals. Ethical approval was obtained from the Yeditepe University Local Ethics Committee for Animal Experiments (Project Protocol No: 2025-11).

Male Sprague-Dawley rats (8–12 weeks old, 350–400 g) were housed under standard light/dark conditions and provided ad libitum access to a standard diet (6% fat content). The animals (n = 20) were randomly divided into four experimental groups:

- Group 1 (G1): No transplantation, without static magnetic field (SMF) (n = 4);
- Group 2 (G2): No transplantation, with SMF (n = 4);
- Group 3 (G3): Transplantation without SMF (n = 6);
- Group 4 (G4): Transplantation with SMF (n = 6).

**Figure 1:**
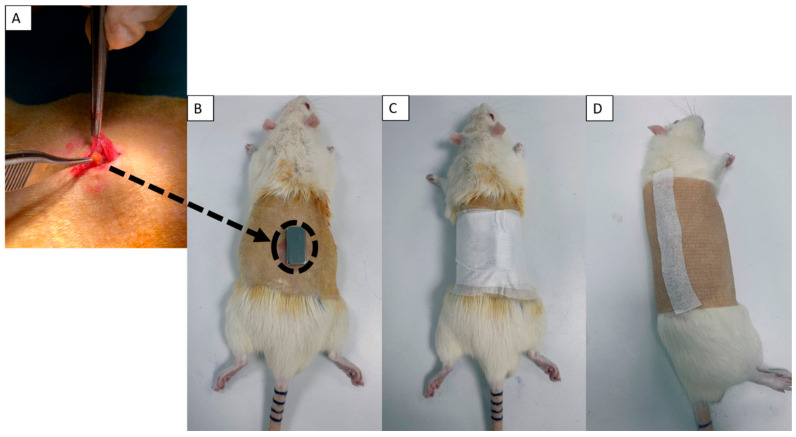
(**A**) Xenotransplantation of hyperplastic human parathyroid tissue into Sprague-Dawley rats; (**B**) surgical localization and fixation of N38 neodymium magnets for continuous SMF in Groups 2 and 4; (**C**,**D**) application of sterile hypoallergenic stabilization patches and protective bandages in Groups 2, 3, and 4.

Prior to surgery, rats were weighed and anesthetized using intraperitoneal (IP) injections of Ketasol (Richter Pharma AG, Wels, Austria) (75 mg/kg, 10%) and Rompun (Bayer AG, Leverkusen, Germany) (0.5 mg/kg, 2%). The dorsal area was shaved, and 0.5 mL of pre-op peripheral blood was collected from the jugular vein. All surgical procedures were performed in the prone position. The skin was disinfected with Baticonol (10% sterile antiseptic solution, Dermosept, Izmir, Turkey), and a 3 cm longitudinal incision was made along the midline of the dorsal surface. For transplantation groups (G3 and G4), individual parathyroid tissue grafts (~3 mm^3^) were implanted intramuscularly beneath the skin fascia between two dorsal muscles, maintaining a 1 cm spacing (Figure 1A). The incision was closed with 4-0 silk sutures and retreated with Betadine. To prevent ocular infections, oxytetracycline (Terramycin 200, Zoetis Inc., Parsippany, NJ, USA) was applied to the rats’ eyes during surgery. After transplantation, rats received intramuscular injections of Cefamezin (1000 mg, Sanofi, Paris, France) and Anaflex (0.5%, Hektaş Ticaret T.A.Ş., Gebze, Kocaeli, Turkey) via the biceps femoris to prevent infection and minimize inflammation. For static magnetic field application (G2 and G4), sterile N38 neodymium magnets (15 × 10 × 5 mm) were placed directly above the graft site (Figure 1B) and secured with sterile hypoallergenic patches and bandages (Figure 1C,D). Patches and bandages were also applied to non-SMF groups (excluding G1) to standardize the physical conditions. These dressings were replaced on days 3, 7, 14, 21, and 28 to minimize pressure buildup and allow for skin inspection. All animals were housed individually in cages constructed from non-magnetic materials. On day 30, rats were anesthetized and euthanized by cervical dislocation. A total of 0.5 mL of post-op peripheral blood was collected via intracardiac puncture. Blood samples were incubated at room temperature (1 h, in the dark) and centrifuged at 4000 revolutions per minute (RPM) for 10 min at 4 °C using serum separator tubes (Greiner Bio-One GmbH, Kremsmünster, Austria, Cat# 450533, Lot: A21023JT). The isolated serum was transferred into Eppendorf tubes and stored at −80 °C until further analysis of systemic PTH levels and cytokine profiles. To mitigate potential mechanical pressure from the magnet apparatus, a rigorous daily care protocol was implemented for the first 14 days. This included brief anesthesia for bandage removal, sterile saline cleaning of the dorsal site, and localized massage to ensure tissue perfusion. These measures ensured that while localized dermal adaptation occurred, no systemic stress or weight loss was induced, preserving the integrity of the physiological data.

### 2.4. Inflammatory Cytokine Assay

Serum cytokine levels were measured before transplantation and after 30 days to evaluate systemic inflammatory responses. The following cytokines were analyzed: IL-1α, IL-1β, IL-6, IL-10, IL-12p70, IL-17A, IL-18, IL-33, CXCL1/KC, CCL2/MCP-1, GM-CSF, IFN-γ, and TNF-α.

Measurements were performed using the LEGENDplex™ Rat Inflammation Panel (13-plex) (BioLegend, San Diego, CA, USA, Cat# 740401, Lot: B314752), following the manufacturer’s instructions. Fluorescence signals were acquired on a flow cytometer (CytoFLEX, Beckman Coulter Inc., Brea, CA, USA) and analyzed using the LEGENDplex™ Data Analysis Software Suite (Version 2025-05-01). Statistical significance was defined as * *p* < 0.05, ** *p* < 0.01, *** *p* < 0.001, and **** *p* < 0.0001. For clarity, a Relative Fold Intensity (RFI) value exceeding 1.3 was classified as a significant increase, and a value below 0.85 was classified as a significant decrease.

### 2.5. PTH Measurement

PTH levels were measured in both serum and tissue samples to evaluate graft functionality and systemic hormonal changes. Pre-op (Day 1) and post-op (Day 30) peripheral blood samples were collected from each rat. Serum was isolated by incubating the blood at room temperature (1 h, in the dark), followed by centrifugation at 4000 RPM for 10 min at 4 °C using serum separator tubes. The resulting serum was transferred into microcentrifuge tubes and stored at −80 °C until analysis. Before PTH quantification, thawed samples were centrifuged at 10,000× *g* for 10 min. Supernatants were used for systemic PTH analysis (pg/mL) to assess pre/post-op differences and the effect of static magnetic field exposure.

Post-op graft tissues from G3 and G4 were collected as experimental samples. For control samples, muscle and fat tissues from the graft implantation region were harvested from G1 and G2. All samples were transported to the laboratory in harvest medium and incubated for 3 h to remove contaminants. Tissues were then transferred to complete culture medium (DMEM-High Glucose (HG) supplemented with 10% fetal bovine serum and 1% penicillin/streptomycin) and incubated for 24 h. After incubation, 1 mL of culture medium from each well was collected and centrifuged at 10,000× *g* for 10 min. Supernatants were used to measure secreted PTH concentrations (pg/mL), serving as a functional indicator of graft activity.

Background absorbance from blank wells was subtracted from each sample’s measurement. PTH levels were quantified using a human PTH ELISA kit (Abcam plc, Cambridge, UK, Cat# ab230931, Lot: P5960) according to the manufacturer’s instructions. All measurements were performed in triplicate. Following PTH analysis, tissue samples were fixed for subsequent histological examination.

### 2.6. Histology

Transplanted parathyroid tissues from G3 and G4 were maintained in harvest medium and incubated individually in well plates at 37 °C with 5% CO_2_ for 24 h to monitor PTH secretion prior to histological processing.

For histological examination, samples were fixed in 10% neutral-buffered formalin at room temperature (RT) for 24 h, then transferred to the pathology department. To ensure objective evaluation and mitigate observer bias, all histological and IHC assessments—including the semi-quantitative scoring of immune cell infiltration (0–3 scale)—were performed by pathologists blinded to the experimental group assignments. Following standard protocols, tissues were processed and embedded in paraffin wax. Sections (4 µm thick) were prepared for hematoxylin and eosin (H&E) staining. Immunohistochemistry (IHC) was performed on these sections using the following primary antibodies; anti-PTH Mouse Recombinant Monoclonal Antibody (Abcam, Cat# ab234415) to identify parathyroid cells, anti-CD68 Mouse Monoclonal Antibody (Abcam, Cat# ab201340) for macrophages, anti-CD20 Rabbit Monoclonal Antibody (Abcam, Cat# ab64088) for B-lymphocytes, anti-CD3 Mouse Monoclonal Antibody (Abcam, Cat# ab17143) for T-lymphocytes. IHC assays were performed using an EnVision FLEX visualization system (Agilent Technologies, Santa Clara, CA, USA) and an Autostainer Link 48 system (Dako, Agilent Technologies, Glostrup, Denmark). In accordance with the instructions of the manufacturer, positive and negative controls were used.

### 2.7. Statistical Analysis

Data were analyzed using GraphPad Prism 9 software (GraphPad Software, San Diego, CA, USA) (Version 9.0.2). A two-way ANOVA was employed to evaluate statistical differences between groups. Tukey’s post hoc test was applied only in cases where the ANOVA indicated statistically significant differences (*p* < 0.05). Statistical significance was defined as * *p* < 0.05, ** *p* < 0.01, *** *p* < 0.001, and **** *p* < 0.0001, with a minimum of three biological replicates per group (*n*  ≥  3).

## 3. Results

This study was initiated to rigorously test the application of a moderate-intensity SMF as a non-pharmacological immune modulator following parathyroid tissue transplantation. Cryopreserved human parathyroid tissue was meticulously processed, sectioned into small fragments (~3 mm^3^), and implanted into an animal model including two control groups (Groups 1 and 2) and two experimental groups (Groups 3 and 4). To establish accurate functional baseline parameters, pre-op (Day 1) serum samples were collected from the jugular vena cava of all animals, allowing for comparative analysis against post-op results for PTH levels.

The post-op analysis conducted at Day 30 was comprehensively evaluated for functional, immunological, and structural outcomes. Functional endpoints included a final measurement of serum PTH levels, as well as assessment of PTH content in the ex vivo transplant tissue and adjacent control tissues (muscle and fat). The immune response was thoroughly evaluated via serum profiles of 14 key inflammatory, regulatory, and chemoattractant cytokines (IL-1α, IL-1β, IL-6, IL-10, IL-12p70, IL-17A, IL-18, IL-33, CXCL1/KC, CCL2/MCP-1, GM-CSF, IFN-γ, and TNF-α). Finally, histological analysis was performed, combining H&E staining for morphological assessment with IHC staining to characterize the remaining functional tissue (PTH) and the composition of the immune infiltrate (CD68 for macrophages, CD20 for B-cells, and CD3 for T-cells).

### 3.1. PTH Measurement

**Figure 2:**
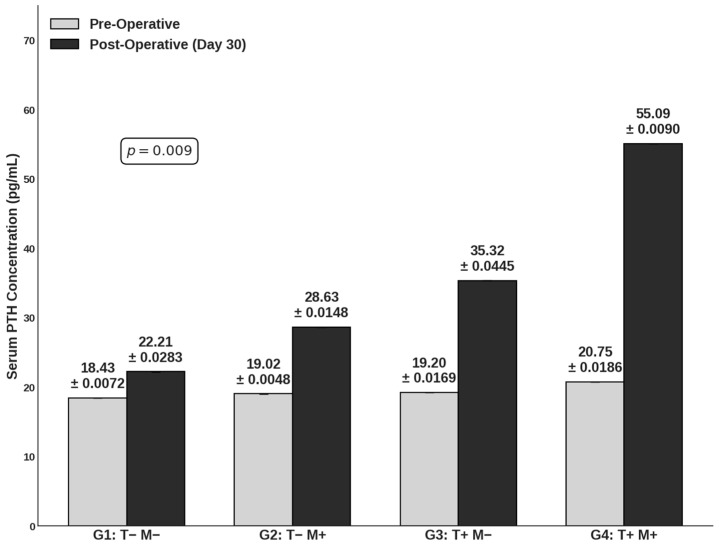
Systemic functional recovery and serum PTH levels following parathyroid xenotransplantation. Bars represent mean serum parathormone (PTH) concentrations (pg/mL) measured at baseline (Pre-Operative; light gray) and at day 30 (Post-Operative; dark gray) across the study population (n = 4 for G1 and G2, n = 6 for G3 and G4). Experimental cohorts are defined as G1: Control (T− M−); G2: SMF exposure only (T− M+); G3: Transplantation only (T+ M−); and G4: SMF-assisted transplantation (T+ M+). T denotes the xenotransplantation of hyperplastic human parathyroid tissue; M denotes continuous 30-day exposure to a moderate-intensity static magnetic field (200–250 mT). Data are presented as mean ± standard deviation (SD). For data transparency, exact mean ± SD values are provided as text annotations above each bar, particularly where SD values are smaller than the bar border weight. Statistical analysis revealed a significance of *p* = 0.009 between the experimental groups at Day 30.

Serum PTH concentrations were quantified in both serum and retrieved tissue samples to evaluate functional graft viability and the modulatory potential of the SMF. Longitudinal analysis revealed that serum PTH concentrations at Day 30 (post-op) were consistently elevated compared to pre-operative baselines. Critically, the transplantation cohorts (Groups 3 and 4) exhibited markedly higher post-operative serum PTH levels compared to the non-transplantation controls (Groups 1 and 2), confirming the successful engraftment and endocrine activity of the human parathyroid xenografts (*p* = 0.009) (Figure 2). Notably, the application of the SMF appeared to potentiate systemic hormone recovery. Group 4 demonstrated the highest mean serum PTH concentration (55.09 pg/mL), substantially exceeding that of Group 3 (35.32 pg/mL) as well as Group 1 (22.21 pg/mL) and Group 2 (28.63 pg/mL). These findings suggest that while the xenograft drives the restoration of hormonal function, the SMF application significantly enhances the magnitude of systemic PTH release.

**Figure 3:**
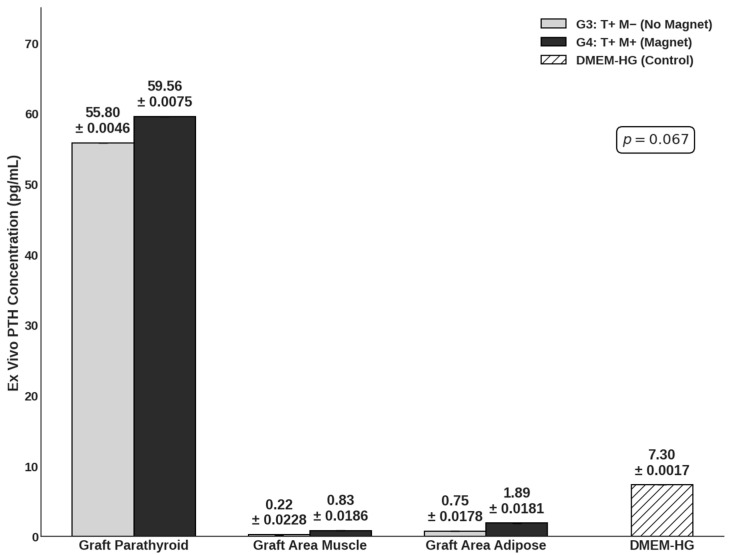
Ex vivo PTH secretory capacity of graft and surrounding tissues. Concentrations of PTH (pg/mL) were measured in the supernatant of harvested tissues following a 24 h incubation period. The graph compares Group 3 (transplantation only; light gray; n = 6) and Group 4 (SMF-assisted transplantation; dark gray; n = 6) across the graft parathyroid, adjacent muscle, and adipose tissues. DMEM-HG (white/hatched) serves as the medium control. Data are presented as mean ± SD. A *p*-value of *p* = 0.067 was observed between the parathyroid graft groups, indicating that secretory function was maintained in SMF-exposed grafts (200–250 mT) compared to non-exposed grafts.

To validate the graft as the source of functional hormone, ex vivo tissue samples were cultured for 24 h (Figure 3). PTH concentrations measured within the parathyroid graft tissue supernatants (Group 3: 55.80 pg/mL; Group 4: 59.56 pg/mL) were significantly higher than those measured in the surrounding control muscle (0.22–0.83 pg/mL) and adipose tissues (0.75–1.89 pg/mL). Crucially, the ex vivo secretory capacity was comparable between the Group 3 and Group 4 groups. This indicates that the moderate-intensity SMF did not negatively alter the intrinsic viability or fundamental secretory mechanics of the parathyroid cells. These findings were also supported with in vitro measurements for both viability (Table S1) and functionality (Table S2). Collectively, these data indicate that the enhanced systemic PTH levels observed in vivo in Group 4 likely result from improved graft survival and integration—potentially mediated by the favorable immunomodulation described earlier—rather than a direct alteration of cellular secretory kinetics.

### 3.2. Histology

**Figure 4:**
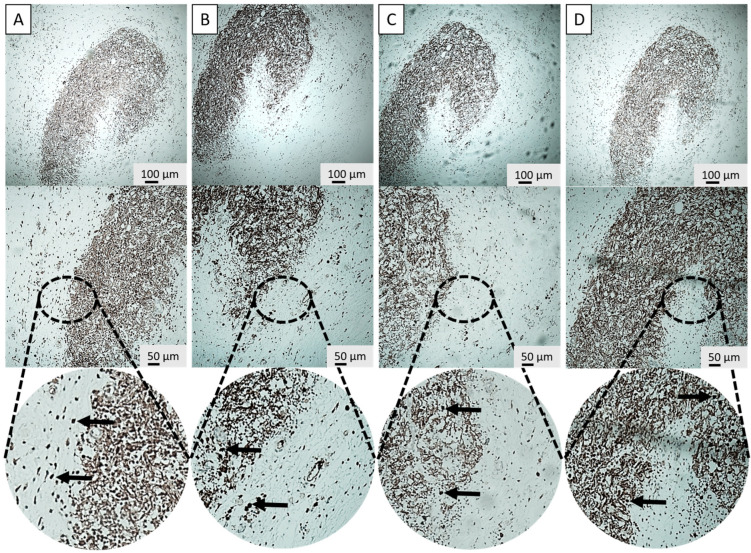
Characterization of immune infiltration in non-SMF grafts (group 3). Immunohistochemistry (IHC) staining of retrieved Group 3 (without SMF) parathyroid grafts displaying diffuse infiltration of CD3 (T-lymphocyte) (**A**), CD20 (B-lymphocyte) (**B**), CD68 (macrophage) (**C**) within the graft parenchyma. (**D**) Positive PTH (parathormone) staining confirms hormonal identity. Magnifications: 10× (upper) and 20× (lower) for each staining. Arrow: Positive staining.

**Figure 5:**
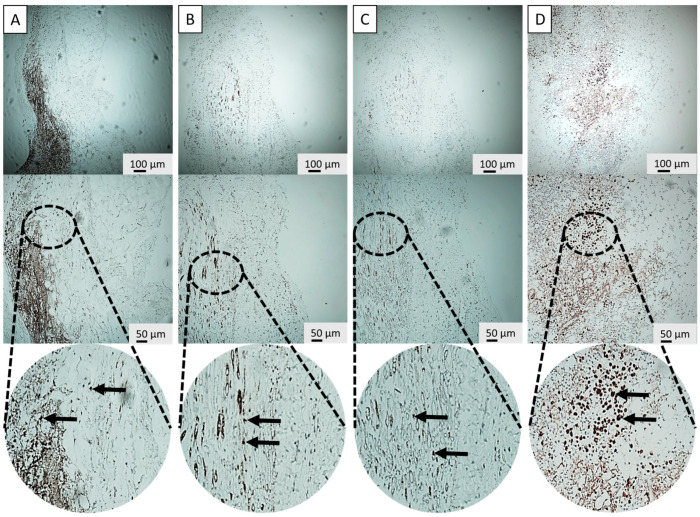
Spatial sequestration of immune cells in SMF-assisted grafts (Group 4). Immunohistochemistry (IHC) staining of retrieved Group 4 (with SMF) parathyroid grafts for CD3 (T-lymphocyte) (**A**), CD20 (B-lymphocyte) (**B**), CD68 (macrophage) (**C**) reveals a marked spatial redirection, with immune cells sequestered to the graft periphery rather than infiltrating the tissue. (**D**) PTH (parathormone) staining confirms preserved graft viability and function. Magnifications: 10× (upper) and 20× (lower) for each staining. Arrow: Positive staining.

IHC and H&E stainings were performed on the retrieved parathyroid grafts from the transplantation groups (G3 and G4) to structurally investigate the host immune response with and without the application of the SMF. Key immune markers included CD3, CD20, and CD68 to specifically identify T-lymphocytes, B-lymphocytes, and macrophages, respectively (Figure 4A–C and Figure 5A–C). Furthermore, PTH staining was used to visually confirm the presence and functional viability of the graft parenchyma, indicated by characteristic brown cytoplasmic staining (Figure 4D and Figure 5D). As Groups 1 and 2 did not receive implants, Group 3 (G3: T+ M−) served as the primary negative control for IHC staining comparisons regarding graft infiltration.

**Figure 6:**
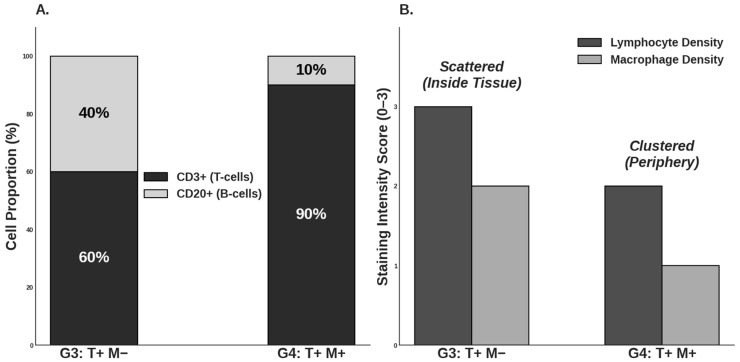
Immunohistochemical (IHC) profiling of the graft immune microenvironment. (**A**) Comparative analysis of lymphocyte subpopulations showing the percentage of CD3+ (T-cells) and CD20+ (B-cells) within the graft area (n = 6 per group). A shift toward a CD3-dominant profile is observed in the SMF-assisted group (G4). (**B**) Semi-quantitative density scores (0–3) for lymphocyte and macrophage infiltration in the transplanted groups. Due to the semi-quantitative nature of the assessment, Panel B displays representative intensity scores based on a discrete 0–3 scale, which precludes the use of standard deviation (SD) bars. Qualitative morphological assessments (scattered vs. clustered) and spatial distribution (inside vs. periphery) are annotated to describe the infiltration pattern. As G1 and G2 (n = 4) served as non-transplant controls and lacked implants, IHC analysis focused on the graft area of G3 and G4. All analyzed graft sections remained positive for PTH expression, confirming maintained hormonal identity during the immune response.

The histological analysis revealed distinct differences in immune cell infiltration patterns between the groups, preliminary supported via in vitro results (Figure S1). In Group 3, macrophage, T- and B-lymphocyte distribution was scattered within the graft, and a higher overall lymphocyte count was detected within the graft area. Essentially, these lymphocytes were often found intermingled with the parathyroid cells, indicating direct contact and immune recognition (Figure 6).

In stark contrast, Group 4 demonstrated a significantly different and more favorable pattern of immune cell distribution. The total number of lymphocytes and macrophages within the graft area was substantially reduced compared to G3. Moreover, the remaining lymphocytes exhibited a highly localized distribution and, critically, were positioned near the parathyroid graft but not intermixed with it (Figure 6).

### 3.3. Cytokine Measurement

**Figure 7:**
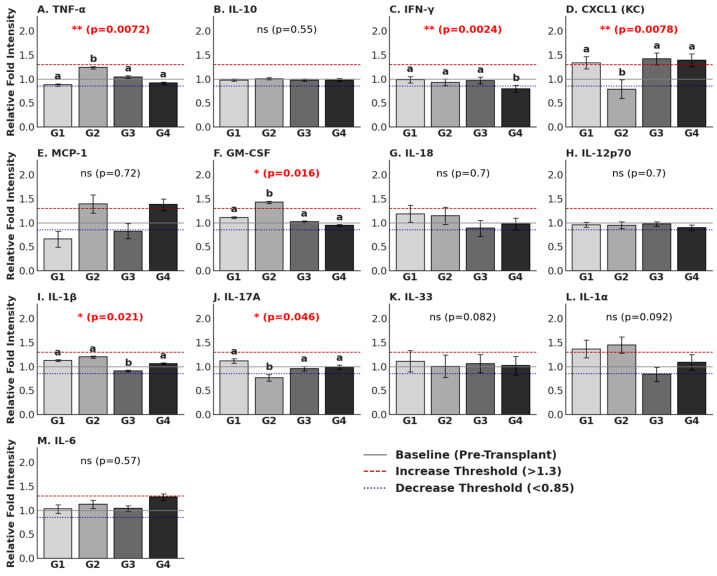
Comparative Analysis of Post-Operative Systemic Cytokine Profiles. Relative Fold Intensity (RFI) normalized to matched pre-operative baseline levels (Baseline RFI = 1.0). (**A**–**M**) Bar charts display the differential expression of 13 cytokines across the four experimental cohorts (n = 4 for G1 and G2, n = 6 for G3 and G4): G1: Control; G2: SMF only (200–250 mT); G3: Transplantation only; and G4: SMF-assisted transplantation (200–250 mT). Each bar represents the mean RFI, with error bars denoting the Standard Deviation (SD) to illustrate data variability. Visual reference thresholds are provided for context: the solid gray line (y = 1.0) represents the pre-operative baseline level, while the dashed red line (y = 1.3) and dotted blue line (y = 0.85) denote the upper and lower thresholds for substantial fold change, respectively. Global *p*-values are indicated at the top of each panel, with significance levels denoted as * *p* < 0.05, ** *p* < 0.01; ns: not significant. For cytokines demonstrating significant global differences, a compact lettering system (a, b) is utilized to indicate specific differences between cohorts; bars that do not share a common letter are significantly different (*p* < 0.05) based on post hoc analysis.

Post-operative serum cytokine levels were assessed on Day 30 across all experimental groups to determine the impact of the static magnetic field (SMF) and the transplantation procedure on the systemic immune response. Relative Fold Intensity (RFI) values were normalized against pre-op baseline levels (RFI = 1.0). An RFI value exceeding 1.3 was classified as a significant increase (indicated as dashed lines in Figure 7), and a value below 0.85 was classified as a significant decrease (indicated as dotted lines).

Comparative group analysis between pre-operative and post-operative samples revealed distinct longitudinal shifts. A significant increase was observed in CXCL1 (KC) (Groups 3 and 4), MCP-1 (Groups 2 and 4), GM-CSF (Group 2), and IL-1α (Groups 1 and 2) (*p* < 0.05). Conversely, serum cytokine levels of CXCL1 (KC) (Group 2), MCP-1 (Group 1), and IL-17A (Group 2) were found to be significantly decreased (*p* < 0.05) (Figure 7).

To further isolate the effects of the novel method, a cross-sectional statistical analysis was performed comparing the four experimental groups: sham/control (Group 1), magnet-only control (Group 2), transplantation only (Group 3), and novel transplantation with magnet (Group 4). Significant inter-group differences were observed in key pro-inflammatory markers. First of all, TNF-α (*p* = 0.0072), while the Group 2 exhibited elevated levels (RFI: 1.24), the Group 4 maintained levels (RFI: 0.92) comparable to the Group 1 (RFI: 0.88). This suggests that the application of the SMF in the context of xenotransplantation does not exacerbate systemic TNF-α levels. Secondly, for GM-CSF (*p* = 0.016), a similar trend was observed; Group 2 showed a pronounced spike (RFI: 1.43), whereas Group 4 displayed a suppressed profile (RFI: 0.95), effectively mitigating the inflammatory increase seen in the magnet-only group. For IL-1β (*p* = 0.021), expression differed significantly across cohorts, with Group 3 showing the lowest expression (RFI: 0.91) compared to the slight elevation observed in Group 2 (RFI: 1.20). As a crucial finding, IFN-γ (*p* = 0.0024) in Group 4 exhibited the lowest systemic IFN-γ levels (RFI: 0.80) among all cohorts, significantly lower than both Group 1 (RFI: 0.99) and Group 3 (RFI: 0.97). This reduction indicates a potential immunomodulatory effect of the novel method in dampening the Th1 response associated with graft rejection. On the other hand, CXCL1 (KC) (*p* = 0.0078) levels in both transplantation groups displayed elevated levels (Group 3: 1.42 RFI; Group 4: 1.39 RFI), distinct from the suppression observed in Group 2 (RFI: 0.79). This suggests that CXCL1 upregulation is driven by the transplantation biology rather than the magnetic field alone. Lastly, IL-17A (*p* = 0.046) levels were significantly lower in Group 2 (RFI: 0.77) compared to the baseline-like stability observed in Group 4 (RFI: 0.99) and Group 1 (RFI: 1.12). As non-significant markers, no statistically significant differences between groups were found for IL-6 (*p* = 0.57), IL-10 (*p* = 0.55), MCP-1 (*p* = 0.72), or IL-12p70 (*p* = 0.70) at the post-operative time point, indicating these markers had stabilized across all conditions by Day 30.

## 4. Discussion

The management of chronic hypoparathyroidism presents a significant clinical challenge, often requiring lifelong calcium and active vitamin D supplementation, which carries risks of hypercalciuria and nephrocalcinosis. While parathyroid allotransplantation is a promising curative approach, its wide clinical adoption is fundamentally limited by the mandatory requirement for chronic, systemic immunosuppression, leading to complications like increased risk of infection, malignancy, and generalized toxicity [46]. The objective of this study was to validate a novel, non-pharmacological strategy employing a moderate-intensity SMF to specifically modulate the host immune response in vivo following parathyroid tissue transplantation. The results from the animal model provided compelling functional and mechanistic evidence supporting the successful and highly localized application of this SMF-assisted immune separation technique.

A foundational consideration when introducing any physical field into the clinical setting is patient safety. Magnetic fields are unique in their ability to propagate through biological tissues without significant attenuation or impedance, overcoming the limitations faced by electric field therapies. As demonstrated by the robust clinical precedent set by Magnetic Resonance Imaging (MRI) [47], even strong magnetic fields are widely accepted as safe, primarily due to the low magnetic susceptibility of human tissues and the negligible presence of ferromagnetic material within the body [48,49]. Our preceding in vitro studies confirmed that moderate-intensity SMF exposure was non-toxic, preserving the viability, proliferation, and genetic integrity of parathyroid cells.

The in vivo PTH measurement results provide the definitive functional correlate to the safety profile established by the cytokine analysis. By demonstrating not only the preservation but also the significant enhancement of xenograft function, we confirmed the therapeutic efficacy of the method. Specifically, the post-operative serum PTH concentrations in Group 4 (55.09 pg/mL) were substantially higher than those in Group 3 (35.32 pg/mL). This profound elevation, far exceeding the non-transplant controls, serves as a robust indicator of successful graft integration and sustained endocrine activity. It suggests that the moderate-intensity SMF fosters a transplant microenvironment that is not merely permissive for survival but one that actively potentiates systemic functional recovery by mitigating subclinical immune-mediated graft loss.

While hyperplastic human parathyroid tissue was utilized, it represents the most clinically relevant source for transplantation studies. Our ex vivo findings confirmed that the secretory kinetics remained stable and were not artificially altered by the SMF, supporting the validity of using this tissue to model hormonal recovery. Specifically, the ex vivo PTH analysis provided the mechanistic insight necessary to interpret this systemic enhancement. When retrieved graft tissues were incubated in a controlled environment, the secretory output was comparable between Group 3 (55.80 pg/mL) and Group 4 (59.56 pg/mL). This finding is pivotal; it indicates that the SMF does not artificially stimulate the baseline secretory kinetics of individual parathyroid cells (hypersecretion). Instead, the discrepancy between the ex vivo (equal per unit tissue) and in vivo (superior systemic accumulation) data suggests that the SMF’s primary benefit lies in cytoprotection. By reducing local immune cell infiltration and suppressing rejection-associated cytokines like IFN-γ, the SMF likely preserved a larger viable functional cell mass over the 30-day period. Consequently, the higher systemic PTH levels in Group 4 reflect a larger surviving population of healthy endocrine cells rather than an alteration in cellular physiology. While direct volumetric measurement of the retrieved grafts was precluded by the surrounding host tissue integration and fibrotic reaction, the combination of superior systemic PTH recovery and preserved histological architecture in Group 4 strongly indicates that SMF-mediated immunoprotection results in a higher net viable cell mass compared to non-SMF controls.

The most critical finding of this in vivo investigation was the structural evidence supporting the hypothesized mechanism of spatial immune separation. The histological analysis of the retrieved animal grafts provided robust in vivo validation for this repulsive phenomenon. While flow cytometric analysis provides high-resolution quantification of total immune cell populations, it necessitates the loss of tissue architecture through digestion. In this study, immunohistochemistry was prioritized to preserve the spatial context required to validate the ‘sequestration’ of immune cells to the graft periphery, as the physical localization of the infiltrate is the primary indicator of SMF-mediated immunoprotection. The IHC staining for CD3 (T-lymphocytes), CD20 (B-lymphocytes) and CD68 (macrophages) revealed profound differences in immune infiltration patterns. In the control transplant group (Group 3), T-lymphocytes and macrophages were frequently observed intermingled with the parathyroid cells, often surrounding the graft parenchyma [50]. This pattern is characteristic of early immune recognition and rejection processes, where direct cell-to-cell contact enables antigen presentation and T-cell cytotoxicity [51]. In stark contrast, the SMF-applied Group 4 showed two distinct advantages: first, a significant reduction in overall lymphocyte and macrophage count within the transplant area, and second, a dramatic shift in cellular localization. The remaining immune cells were predominantly localized near the peripheral fibrotic overgrowth but were not intermixed with the viable parathyroid tissue. Localization of immune cells confirms that the magnetic field successfully established a physical immune-privileged zone around the transplant interface. This finding strongly supports the proposed mechanism that the SMF interferes with key cellular processes essential for immune infiltration. The in vitro observations of Jurkat cell migration, as detailed in Figure S1 and Videos S1 and S2, provide a foundational biophysical proof-of-concept for the ‘spatial sequestration’ phenomenon seen in vivo. In these controlled settings, we observed a clear directional shift in lymphocyte positioning when exposed to the 300 mT SMF. This effect likely stems from Lorentz force interactions on membrane ion channels and the induction of diamagnetic torque on the cytoskeleton. These physical forces together appear to impair the cell’s ability to maintain a stable leading edge, effectively repelling them from the graft interface before direct contact can occur. The related mechanism operates on two primary levels: firstly, according to the adhesion dynamics, lymphocyte adhesion is mediated by membrane molecules like integrins (e.g., LFA-1 and VLA-4) binding to their ligands. The applied SMF may alter membrane fluidity and the arrangement of the cytoskeleton (actin polymerization), which is essential for cell shape, motility, and adhesion stability [52]. By reducing the stability of these adhesion points, the magnetic force can more readily overcome the weakened binding strength, promoting separation. Secondly, by affecting the directional motility and polarization, the magnetic force may induce a directional migration analogous to electrotaxis—the movement of cells in an electric field [52,53]. Signaling cascades such as PI3K-Akt, MAPK, and calcium-dependent mechanisms are central to lymphocyte polarization, chemotaxis, and immune function [54]. Disruption of these pathways through magnetic modulation could impair immune cells’ ability to adhere, migrate, or respond to microenvironmental cues—especially when fibrotic growth already limits physical interaction [55,56]. Although T cells are not classically magnetotactic, the interplay between the SMF and intracellular components, particularly charge gradients and cytoskeletal alignment, may induce a directional bias. This effectively promotes the movement of T cells away from the region of SMF influence, thereby actively separating them from the graft.

The assessment of serum cytokine profiles provided critical insights into the systemic immunological consequences of the SMF used in our novel parathyroid xenotransplantation method [57]. A primary concern in using magnetic biomaterials is the potential for a foreign body response and systemic inflammatory toxicity. Our results indicate that the combined application of the SMF with parathyroid tissue (Group 4) successfully mitigates the inflammatory spikes observed when the magnet force is applied.

The distinct cytokine signatures of Group 2 versus Group 4 highlight a crucial biological interaction. The significant elevation of TNF-α (*p* = 0.0072) and GM-CSF (*p* = 0.016) in Group 2 suggests that the magnet only may trigger a localized foreign body reaction, prompting systemic macrophage and granulocyte activation. However, this pro-inflammatory response was absent in Group 4. The presence of the parathyroid graft appears to modulate the host response to the magnet, maintaining TNF-α and GM-CSF levels comparable to Group 1. Perhaps the most significant finding for graft survival was the modulation of IFN-γ. Interferon-gamma is a hallmark cytokine of the Th1 adaptive immune response and a primary driver of acute allograft rejection [58]. In our study, Group 4 exhibited the lowest systemic IFN-γ levels (RFI = 0.80), significantly lower than both Group 1 and Group 3. This suppression suggests that the SMF may exert a specific immunomodulatory effect on T-cell proliferation or differentiation when applied to the graft site. By dampening the Th1 axis, the novel method may create a more tolerogenic systemic environment favorable for long-term graft function, potentially reducing the requirement for heavy systemic immunosuppression. The analysis of chemokines, specifically CXCL1 (KC), allowed us to distinguish between surgical trauma and biomaterial-induced inflammation. CXCL1, a potent neutrophil chemoattractant, was upregulated in both transplantation groups (Groups 3 and 4) regardless of the presence of the magnet, while it was suppressed in the magnet-only group. This indicates that the recruitment of neutrophils is a consequence of the transplantation biology and surgical engraftment process rather than an adverse reaction to the magnetic field itself.

Finally, the stability of broad-spectrum inflammatory markers such as IL-6 (*p* = 0.57) and IL-10 (*p* = 0.55) across all groups confirms that the novel method does not induce a systemic “cytokine storm” or generalized acute-phase response. The cytokine changes observed were specific and targeted, primarily involving the modulation of rejection-associated pathways (IFN-γ) and local tissue response markers (TNF-α), supporting the systemic safety profile of the SMF-assisted transplantation technique.

In summary, this study demonstrated a significant and clinically actionable step toward developing a non-immunosuppressive long-term therapy for hypoparathyroidism. Our findings established a robust dual-protective mechanism for graft survival. Firstly, the natural fibrotic overgrowth that replaced the hydrogel capsule used in general in vitro models provides a strong physical barrier against direct, large-scale immune invasion, preventing rapid rejection. Secondly, the externally applied moderate-intensity SMF provides the active, dynamic immune modulation necessary to repel or redirect the individual T-lymphocytes and macrophages that breach the primary fibrotic barrier, thereby neutralizing the most critical component of graft rejection. This approach effectively minimizes direct immune cell engagement with the graft, resulting in reduced local inflammation and significantly enhanced post-op functional PTH output in vivo.

While these findings are exceptionally promising, future studies must focus on several critical steps for clinical translation. Optimization is required to determine the optimal magnetic field gradients, dose (duration of application), and configuration of the magnetic poles for maximal migratory and repulsive effect [39,41,43,45]. While the current sample size provided sufficient power to detect significant differences in hormonal and cytokine profiles, the 30-day study duration was intentionally selected to align with the primary clinical assessment milestones for parathyroid transplantation. To overcome the logistical and animal welfare constraints associated with external bandaging and magnet fixation, future studies utilizing biocompatible magnetic implants are recommended to facilitate follow-up durations exceeding 60–90 days. Ultimately, expanding the cohort in future large-animal transplantation settings with these extended follow-up periods remains essential to fully evaluate the long-term sustainability and clinical viability of this unique SMF-assisted immunomodulation strategy.

## 5. Conclusions

This study successfully validates a novel, non-pharmacological strategy for parathyroid allotransplantation by leveraging an externally applied moderate-intensity SMF to modulate the local host immune response in vivo. Our findings demonstrate that this approach achieved spatial immune separation, actively redirecting lymphocyte positioning and dramatically reducing inflammatory cell infiltration at the transplant site. Crucially, this intervention was achieved without compromising parathyroid cell viability or functional PTH secretion, confirming the safety and functional efficacy of the technique.

The use of controlled, physical forces—specifically, magnetically influenced lymphocyte migration—represents a significant conceptual and technological advancement in transplant immunomodulation. By creating a localized, immune-privileged environment, this method addresses a major limitation in current transplantation protocols: the reliance on systemic immunosuppressive drugs or complex invasive encapsulation systems. This dual-action strategy, which combines physical barriers with active immunological redirection, has the potential to maintain long-term graft survival while preserving natural endocrine function.

This technique may pave the way for a next-generation therapeutic option for patients with chronic hypoparathyroidism. To fully realize its clinical potential, future research must prioritize larger animal transplantation settings and employ longer follow-up periods (beyond the 30-day time point) to validate long-term sustainability and ultimate safety. If successful, this approach could lead to a durable, lifelong solution, offering high transplantation success without lifelong medication, thereby significantly improving patient quality of life and reducing healthcare burdens.

## Data Availability

The original contributions presented in this study are included in the article/Supplementary Materials. No datasets were generated or analyzed during the current study. Should any raw data files be needed in another format, they are available from the corresponding author upon reasonable request.

## Supplementary Materials

The following supporting information can be downloaded at: https://www.mdpi.com/article/10.3390/cells15070600/s1. Figure S1: Live-cell imaging of Jurkat cells co-cultured with encapsulated parathyroid cells; Table S1: In vitro cell viability results of parathyroid cell-containing groups; Table S2: In vitro PTH concentrations of parathyroid cell-containing groups; Video S1: Live-cell imaging of Jurkat cells co-cultured with encapsulated parathyroid cells without magnetic field; Video S2: Live-cell imaging of Jurkat cells co-cultured with encapsulated parathyroid cells with magnetic field.

## Abbreviations

The following abbreviations are used in this manuscript:

µm	Micrometer
µT	Microtesla
ANOVA	Analysis of Variance
Ca	Calcium
CCL2	Chemokine Ligand 2
CD	Cluster of Differentiation
CO_2_	Carbon Dioxide
CXCL1(KC)	Chemokine Ligand 1
DC	Direct Electric Current
DMEM-HG	Dulbecco’s Modified Eagle Medium-High Glucose
FBS	Fetal Bovine Serum
GM-CSF	Granulocyte-Macrophage Colony-Stimulating Factor
H&E	Hematoxylin and Eosin
IFN-gamma	Interferon-gamma
IHC	Immunohistochemistry
IL	Interleukin
LFA-1	Lymphocyte Function-Associated Antigen-1
MAPK	Mitogen-Activated Protein Kinase
MFs	Magnetic Fields
mg	Milligram
MHC	Major Histocompatibility Complex
mL	Milliliter
MRI	Magnetic Resonance Imaging
mT	Millitesla
n	Sample Size
*p*	P-value (Statistical Significance)
P/S	Penicillin/Streptomycin
PBS	Phosphate-Buffered Saline
pg	Picogram
PI3K-Akt	Phosphoinositide 3-kinase-Protein Kinase B
Pre/Post-op	Pre/Post-operative
PTH	Parathyroid Hormone
RFI	Relative Fold Intensity
RPM	Revolutions per Minute
RT	Room Temperature
SD	standard deviation
SMF	Static Magnetic Field
T	Tesla
Th1/2	T Helper Type 1/2
TNF-alpha	Tumor Necrosis Factor-alpha
