# Supplementary material for: Static Magnetic Field-Mediated Parathyroid Xenotransplantation Modulates Lymphocyte Migration: A Potential Immunosuppression-Free Long-Term Treatment for Hypoparathyroidism
Source: Cells. 2026 Mar 28;15(7):600. doi: 10.3390/cells15070600 (PMC13072275; doi:10.3390/cells15070600)

Figure S1. Live-cell imaging of Jurkat cells co-cultured with encapsulated parathyroid cell-containing groups without magnetic field (**A**, **B**) and with magnetic field (**C**, **D**) surrounding the alginate capsule after 24 hours (**A**, **C**) and 72 hours (**B**, **D**) of incubation, magnification: X 2.5. Initial cell seeding/encapsulating density was 100,000 cells for each well and microcapsule.

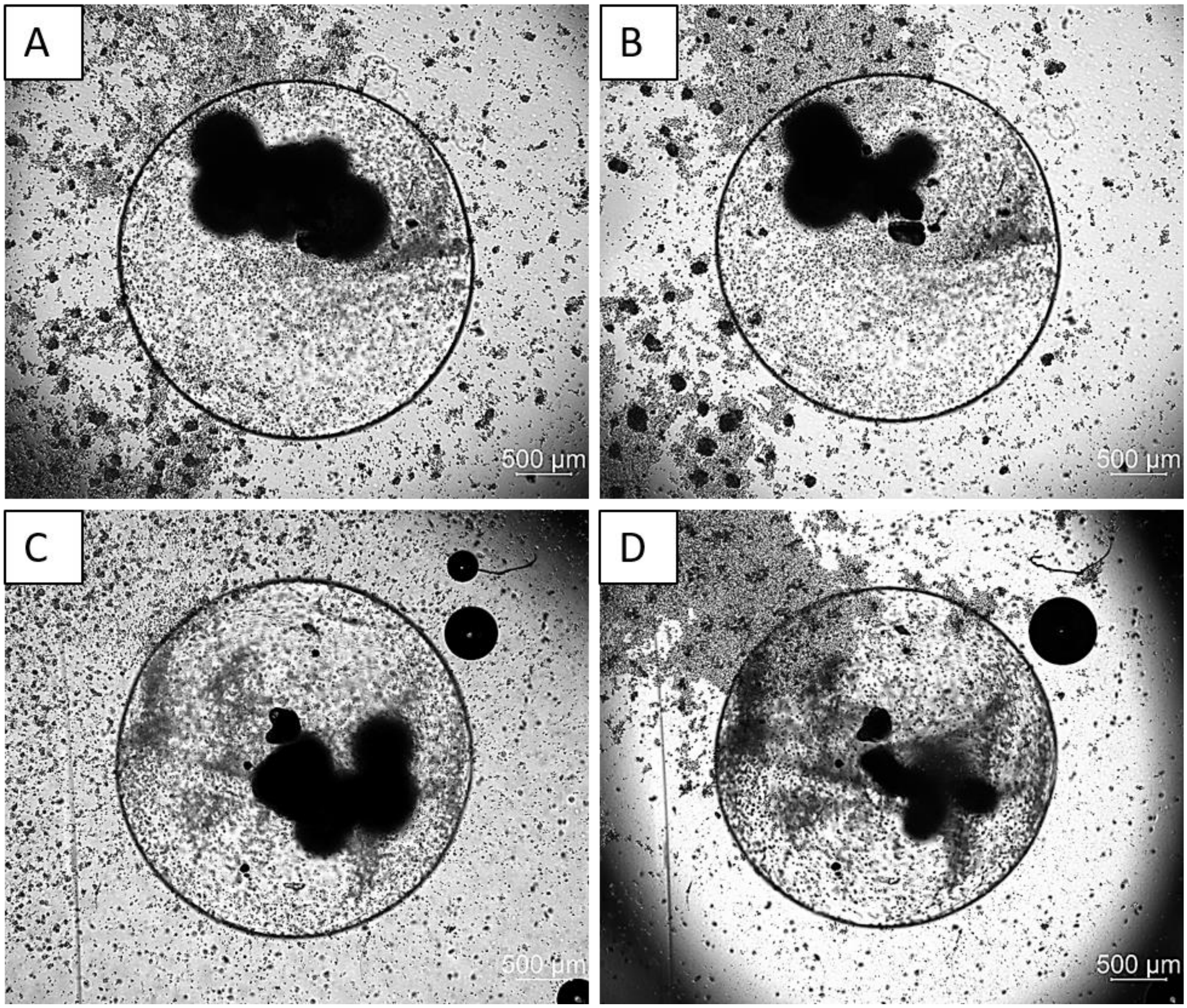

Supplement: Supplementary file 1 [file cells-15-00600-s001.zip › Figure S1 - Live-cell imaging of Jurkat cells co-cultured with encapsulated parathyroid cells..pdf]
