# Supplementary material for: Static Magnetic Field-Mediated Parathyroid Xenotransplantation Modulates Lymphocyte Migration: A Potential Immunosuppression-Free Long-Term Treatment for Hypoparathyroidism
Source: Cells. 2026 Mar 28;15(7):600. doi: 10.3390/cells15070600 (PMC13072275; doi:10.3390/cells15070600)
Supplement: Supplementary file 1 [file cells-15-00600-s001.zip › Table S1 - In vitro cell viability results of parathyroid cell-containing groups..pdf]

Table S1. *In vitro* cell viability results of parathyroid cell-containing groups by Live/Dead Assay ($p < 0.01$) after 24 and 72 hours of incubation. Initial cell seeding/encapsulating density was 100,000 cells for each well and microcapsule.

Groups	Magnetic Field	Exposure Time (h)	Live Cells (%)
Encapsulated Parathyroid Cells	-	24 / 72	81 / 83
Encapsulated Parathyroid Cells	+	24 / 72	80.2 /92
Encapsulated Parathyroid Cells + Jurkat Cells	-	24 / 72	85 / 82
Encapsulated Parathyroid Cells + Jurkat Cells	+	24 / 72	88 / 79.4
