# Supplementary material for: Static Magnetic Field-Mediated Parathyroid Xenotransplantation Modulates Lymphocyte Migration: A Potential Immunosuppression-Free Long-Term Treatment for Hypoparathyroidism
Source: Cells. 2026 Mar 28;15(7):600. doi: 10.3390/cells15070600 (PMC13072275; doi:10.3390/cells15070600)
Supplement: Supplementary file 1 [file cells-15-00600-s001.zip › Table S2 - In vitro PTH concentrations of parathyroid cell-containing groups..pdf]

Table S2. *In vitro* PTH concentrations of parathyroid cell-containing groups ($p > 0.05$) after 24, 48 and 72 hours of incubation. Initial cell seeding/encapsulating density was 100,000 cells for each well and microcapsule.

Groups	Magnetic Field	Incubation Period (h)	Concentration (pg/mL)
Encapsulated Parathyroid Cells	-	24 / 48 / 72	362.0 / 257.60 / 304.62
Encapsulated Parathyroid Cells	+	24 / 48 / 72	327.83 / 269.98 / 329.50
Encapsulated Parathyroid Cells + Jurkat Cells	-	24 / 48 / 72	287.60 / 434.86 / 315.57
Encapsulated Parathyroid Cells + Jurkat Cells	+	24 / 48 / 72	282.83 / 247.83 / 307.12
